# Burnout, ethical climate and work organization in covid-19 intensive care units: mixed method study

**DOI:** 10.1590/0034-7167-2022-0684

**Published:** 2023-12-04

**Authors:** Ademir Jones Antunes Dorneles, Graziele de Lima Dalmolin, Edison Luiz Devos Barlem, Rosemary Silva da Silveira, Rafaela Andolhe, Silviamar Camponogara, Tânia Solange Bosi de Souza Magnago, Valdecir Zavarese da Costa

**Affiliations:** I Universidade Federal de Santa Maria. Santa Maria, Rio Grande do Sul, Brazil; II Universidade Federal do Rio Grande. Rio Grande, Rio Grande do Sul, Brazil

**Keywords:** Burnout, Occupational, COVID-19, Intensive Care Units, Nursing, Occupational Health, Agotamiento Profesional, COVID-19, Unidades de Cuidados Intensivos, Enfermería, Salud Laboral, Esgotamento Profissional, Ad26COVS1, Unidades de Terapia Intensiva, Enfermagem, Saúde do Trabalhador.

## Abstract

**Objectives:**

to analyze the association between burnout and the perception of the ethical climate in nursing professionals in the covid-19 Intensive Care Unit and the relationship with the organization of work from the perspective of managers of these units.

**Methods:**

mixed method study conducted in three university hospitals in southern Brazil from December 2021 to March 2022. A cross-sectional study was developed with 110 nursing professionals, followed by an exploratory-descriptive study through semi-structured interviews with six managers. Descriptive and analytical statistics and discursive textual analysis were used.

**Results:**

the prevalence of burnout was 10% and the perception of negative ethical climate was 24.5%. The association between burnout and ethical climate revealed overload and fatigue during working hours, related to tension, fear, and stress that emerged from the consequences of the organization and relations of work in the covid-19 Intensive Care Unit.

**Conclusions:**

there was an association between burnout and ethical climate and elements of the work organization.

## RESUMO

**Objetivos:**

analisar a associação entre *burnout* e percepção do clima ético em profissionais de enfermagem de Unidade de Terapia Intensiva covid-19 e a relação com a organização do trabalho na perspectiva dos gestores dessas unidades.

**Métodos:**

estudo misto realizado em três hospitais universitários no Sul do Brasil, de dezembro de 2021 a março de 2022. Empregou-se estudo transversal com 110 profissionais de enfermagem, seguido de estudo exploratório-descritivo por meio de entrevistas semiestruturadas com seis gestores. Utilizou-se estatística descritiva e analítica, e análise textual discursiva.

**Resultados:**

evidenciou-se 10% de prevalência de *burnout* e 24,5% de percepção do clima ético negativo. A associação entre *burnout* e clima ético revelou sobrecarga e cansaço nas jornadas de trabalho, relacionados à tensão, medo, e estresse, que emergiram das consequências da organização e das relações do trabalho nas UTI covid-19.

**Conclusões:**

verificou-se a associação entre *burnout* e clima ético e elementos da organização do trabalho.

## RESUMEN

**Objetivos:**

analizar la asociación entre el agotamiento profesional y la percepción del clima ético en profesionales de enfermería de la Unidad de Cuidados Intensivos covid-19 y la relación con la organización del trabajo desde la perspectiva de los directivos de estas unidades.

**Métodos:**

estudio de método mixto realizado en tres hospitales universitarios del sur de Brasil entre diciembre de 2021 y marzo de 2022. Se desarrolló un estudio transversal con 110 profesionales de enfermería, seguido de un estudio exploratorio-descriptivo mediante entrevistas semiestructuradas con seis gestores. Se utilizó estadística descriptiva, analítica y análisis textual discursivo.

**Resultados:**

hubo un 10% de prevalencia de agotamiento profesional y un 24,5% de percepción de clima ético negativo. La asociación entre el agotamiento profesional y el clima ético reveló sobrecarga y fatiga durante la jornada laboral, relacionadas con la tensión, el miedo y el estrés que surgieron de las consecuencias de la organización y las relaciones laborales en la Unidad de Cuidados Intensivos covid-19.

**Conclusiones:**

hubo asociación entre agotamiento profesional y clima ético y elementos de la organización del trabajo.

## INTRODUCTION

The whole world faced a great threat in December 2019, when the first cases of covid-19, caused by the coronavirus, began to appear in China. Many events occurred from that moment on, all resulting from this virus with high transmission power between people, difficult to control, which spread rapidly in all parts of the world, constituting a pandemic^(1)^.

In this scenario, people with severe cases of covid-19 needed advanced life support. They were sent to intensive care units that were not prepared nor equipped to care for a large number of patients with cardiac, respiratory and hemodynamic changes. This culminated in the creation of covid-19 intensive care units (covid-19 ICU)^(1-3)^.

In the present scenario, health professionals, specifically nursing professionals, face many challenges and experience different stressors, such as intrapersonal (lack of knowledge about the disease), interpersonal (social relationships) and extrapersonal (work overload)^(4)^. In this process, Nursing, for being a profession that works very closely with patients and their families, was vulnerable to occupational diseases such as burnout, and similarly, subject to the deleterious effects of a negative ethical work climate^(3-4)^.

Burnout is conceptualized as a chronic work syndrome resulting from prolonged exposure to stressors, characterized by Emotional Exhaustion (EE) (loss of energy), Depersonalization (DP) (emotional hardening) and low Professional Accomplishment (PA). This condition produces physical (headache, weakness, dyspnea, weight loss), psychological (feelings of hopelessness, anxiety, indecision, anger, restlessness) and behavioral (reluctance to work, conflicts in the social and work environment) symptoms^(4-5)^.

The ethical climate (EC) is part of the general environment that constitutes the organizational work climate in a hospital or health organization, addressing the collective perception of professionals about the meaning of what is ethically acceptable in the institution^(6)^. It can influence interpersonal relationships, work results, institutional quality, as well as workers’ behavior and culture^(6-7)^.

As for the relationship between EC and burnout variables, a study of Iranian intensive care nurses in the period prior to the pandemic identified a favorable EC, but high scores for frequency and intensity of burnout, to which the authors associated the multifactorial etiology of burnout. That is, although an ICU scenario comprises several stressors, such as the complexity of critical patient care itself, the environment with artificial lighting, noise, alarms, a wide range of procedures and monitoring, the high workload and the fragility of sizing of personnel, the perception of EC can be satisfactory based on the adequate relationship with colleagues, teams and managers^(8)^.

Two literature reviews, one integrative and the other narrative, were carried out to delimit the object of this study. The first, on EC in a hospital environment, was performed in the National Library of Medicine of the United States (PUBMED), Latin American and Caribbean Health Sciences Literature (LILACS), Sci Verse Scopus (SCOPUS) and Web of Science (WoS) databases^(7)^. The second was performed in the Catalog of Theses and Dissertations of the Portal of the Coordination for the Improvement of Higher Education Personnel to learn about trends in scientific production on burnout and nursing in the hospital environment^(9)^. The results showed a gap in scientific production on burnout and EC in the hospital setting, especially in the covid-19 ICU with the nursing population.

The covid-19 pandemic represented a crisis with high psychological suffering for health professionals. In a study of covid-19 ICU physicians was identified a 51.8% prevalence of burnout and association with EC, and data showed greater fragility in the classification of EC in the relationship with administrators^(10)^. In this sense, it is relevant to specifically investigate these variables from the perspective of nursing, and consider the conduct of work organization in that period and place from the perspective of managers.

Thus, this study was designed based on the following research questions: “Is burnout associated with the perception of EC among nursing professionals working in covid-19 ICU?” and “What is the relationship between burnout, the perception of EC and the organization of work in the covid-19 ICU in the perception of managers working in these units?”

## OBJECTIVES

To analyze the association between burnout and the perception of EC in nursing professionals in the covid-19 ICU and the relationship with the organization of work from the perspective of managers of these units.

## METHODS

### Ethical aspects

The national ethics guidelines were respected in the development of the present study, according to Resolution 466/12^(11)^. It was approved by the Research Ethics Committee of the Universidade Federal do Rio Grande (the opinion is attached to this submission) and the Informed Consent was obtained from all study participants electronically.

### Design, study location and period

This is a mixed methods study; a cross-sectional study in the quantitative part (QUAN) and an exploratory-descriptive study in the qualitative part (Qual). The combination of data occurred through the connection, where the secondary findings (qual) had a supporting role to the information from the main database (QUAN). Weight was attributed to the quantitative approach (QUAN+qual), constituting a sequential explanatory strategy (QUAN+qual). The theoretical perspective or implicit “lens” related to burnout and EC was used^(12-13)^. The description of the method was guided by the Strengthening the Reporting of Observational Studies in Epidemiology (STROBE) of the EQUATOR network.

The study was developed in three (H1, H2 and H3) university hospitals (UH) linked to the Brazilian Company of Hospital Services (EBSERH) in the state of Rio Grande do Sul (RS). These UH provide health services to a population of more than 1.2 million inhabitants in RS solely through the Unified Health System (Brazilian SUS). Note that institutions were organized through committees and contingency plans during the pandemic. Managers of hospitals and units got together to develop flows with the purpose of systematizing care, ensuring staff dimensioning and providing greater security in planning and decision-making, both organizational and clinical.

The QUAN stage took place between December 2021 and March 2022 and the Qual stage in the period from May to July 2022, both online.

### Population and sample

The sample in the QUAN stage was defined based on a population of 291 nursing professionals, 81 nurses and 201 nursing technicians. Using a sample calculation formula for a finite population, a minimum number of 102 participants was estimated, of which 29 were nurses and 73 were nursing technicians. In the Qual stage, managers of the covid-19 ICUs were invited. Thus, the population consisted of 110 nursing professionals (QUAN) and six managers (Qual) who worked in the covid-19 ICU of UH, totaling 116 study participants.

### Inclusion and exclusion criteria

In the QUAN stage, the inclusion criteria adopted were being a nurse or nursing technician and having worked in a covid-19 ICU. No minimum time of work in the ICU covid-19 was defined for participation in the study. The convenience sampling technique was used. In the Qual stage, health professionals who acted as managers of the covid-19 ICUs were included.

Nurses, nursing technicians and covid-19 ICU managers who were unavailable during the data collection period were excluded.

### Study protocol

The study protocol was developed in two stages; the first QUAN and the second Qual. The QUAN stage included the application of a sociodemographic and work characterization questionnaire (age, sex, marital status, number of children, schooling, leisure activities, linked UH, professional category, current sector in the UH, work department during the covid-19, nature of the work engagement, time in the profession, time working at the UH, work leave, reason for leave, days off, quitting the job); the Maslach Burnout Inventory (MBI) - HSS version (Human Services Survey) and the Hospital Ethical Climate Survey - Brazilian Version (HECSVB).

Regarding the instruments used in this study, the MBI has 22 questions on a Likert scale ranging from zero - “never” - to four - “daily” - where participants mark the frequency with which they perceive or feel in relation to each question statement. It comprises the following dimensions: EE, consisting of nine questions (1, 2, 3, 6, 8, 13, 14, 16 and 20); DP, consisting of five questions (5, 10, 11, 15 and 22); and PA, consisting of eight questions (4, 7, 9, 12, 17, 18, 19 and 21). High scores in EE and DP, associated with low scores in PA indicate the individual is in burnout^(5,14)^.

The HECSVB has 26 questions divided into five factors: peers (4 items: 1, 10, 18, 23); patients (4 items: 2, 6, 11 and 19); managers (6 items: 3,7,12,15,20 and 24); hospital (6 items: 4, 8, 13, 16, 21, and 25) and physicians (6 items: 5, 9, 14, 17, 22, and 26). The items are related to the ethical issues involved in the work environment and in relationships between nurses and their peers, physicians, patients and management. The instrument consists of a five-point Likert scale with response options ranging from one - “almost never true” to - five - “almost always true”^(6,15)^.

Continuing with the study protocol, in the second stage (Qual), a semi-structured interview script was used. The questions were prepared according to the statistically significant associations (p<0.05) found in the variables in the QUAN stage. The closing criterion for this step was data saturation, as all available participants were invited or included. Excerpts from the qualitative interviews were coded using Indo-Arabic letters and numbers (X1, X2, X3, X4, X5 and X6) in order to preserve the anonymity of participants.

### Analysis of results and statistics

In the QUAN stage of the study, an Excel 2010 spreadsheet was used for the tabulation of data. Afterwards, data analysis was performed in the PASW Statistic^®^ program (Predictive Analytics Software, from SPSS Inc., Chicago, USA) version 18.0 for Windows. Sociodemographic and labor variables were analyzed using descriptive statistics. In the definition of burnout, the cutoff points for EE and DP dimensions were obtained using the 75^th^ percentile, and for the PA dimension, the 25^th^ percentile, which has a reverse score^(5,13,15)^. The dependent variable evaluated was the “presence of burnout”. The EC was evaluated based on the average of factors and dichotomized into positive or negative EC based on the cutoff point of 3.5^(6,15)^. Finally, for the association of EC with burnout, the Mann-Whitney test was used based on the observation of asymmetric data distribution according to the Kolmogorov-Smirnov test. Associations with p<0.05 were adopted as significant.

In the Qual stage of the study, the reports extracted from the interviews were transcribed in Microsoft Word^®^ for Windows. Discursive Textual Analysis (DTA) was superimposed on the group of qualitative results, comprising the steps: disassembling the texts, establishing relationships, capturing the new emergent and the self-organized process^(12-13)^.

In the mixing of results, the QUAN data with statistical significance (p<0.05) extracted from questionnaires were connected side by side with the Qual results aiming at deepening the evidence found, looking for the mixing and interdependence between elements. In this step, the amplification with which the Qual results explained and added information to the QUAN results was analyzed. The mixing of data occurred per connection. In continuity, the two interactive phases originated different classes of data combined around thematic axes of the integrative analysis^(12-13)^.

## RESULTS

Participation of 110 nursing professionals in the study, corresponding to 37.8% of the population. Of these, 53 (48%) were from UH1, 30 (27%) from UH2 and 27 (25%) from UH3. Of these, 29 (26%) were nurses and 81 (74%) nursing technicians; with a median age of 40 years (IQR=5); a median of 12 years (IQR=6) of professional experience in nursing and six years (IQR=6) of experience in the UH (Table 1).

**Table 1:** Result of the associations between dimensions of burnout and factors of the ethical climate in nursing professionals of the university hospitals of the Brazilian Company of Hospital Services in Rio Grande do Sul, Santa Maria, Rio Grande do Sul, Brazil, 2022 (N=110)

	n	Peers			Patients			Managers			Hospital			Physicians			Overall EC		
		Md	IQR	U	Md	IQR	U	Md	IQR	U	Md	IQR	U	Md	IQR	U	Md	IQR	U
EE	Low (77)	3.75	0.63	811.50^*^	3.75	0.75	741.50^*^	3.83	0.75	894.50^*^	3.67	0.67	856.00^*^	3.67	0.67	902.50^*^	3.73	0.64	806.50^*^
	High (33)	4.25	0.88		4.25	0.88		4.15	0.83		4.00	0.58		4.00	1.25		4.08	0.80	
DP	Low (70)	4.00	0.50	916.50^*^	4.00	0.75	898.00^*^	4.00	0.83	842.50^*^	3.83	0.67	930.00^*^	3.92	0.71	885.50^*^	3.96	0.67	849.50^*^
	High (40)	3.75	0.75		3.50	0.69		3.67	0.67		3.67	0.67		3.50	0.67		3.62	0.62	
PA	Low (27)	3.75	0.50	822.50^*^	3.50	0.50	799.00^*^	3.67	0.67	800.50^*^	3.50	0.67	736.50^*^	3.50	0.83	827.50^*^	3.62	0.43	741.50^*^
	High (83)	4.00	0.75		4.00	0.75		4.00	0.67		3.83	0.67		3.83	0.83		3.93	0.67	
SB	Absent (99)	4.00	0.75	322.50^*^	3.75	0.75	311.00^*^	4.00	0.67	331.50^*^	3.83	0.67	284.50^*^	3.83	0.67	219.00^*^	3.85	0.65	263.00^*^
	Present (11)	3.50	0.50		3.50	0.75		3.50	0.67		3.50	1.33		3.17	0.67		3.52	0.83	

EC - Ethical climate; DP - depersonalization; EE - emotional exhaustion; IQR - interquartile range; Md - median; n - number; PA - professional achievement; SB - burnout; significance -

* p < 0.05; U - Mann-Whitney test.

Note that 97 (88.2%) were female, 92 (83.3%) had a partner (marital status), 89 (89.1%) had children, 19 (17.3%) had another job, 46 (41.8%) took time off from work during the pandemic, 32 (29.1%) intended to quit their jobs and 42 (38.2%) were engaged in leisure activities.

In the evaluation of burnout dimensions, 33 (30%) participants with high EE, 40 (36.4%) with high DP and 27 (24.5%) with low PA were found. The analysis of the burnout dimensions evidenced that 11 (10%) nursing professionals were experiencing burnout. Of these, five (17.2%) were nurses and six (7.4%) were nursing technicians.

With regard to EC in the perception of nursing professionals, the following mean values by factors were identified: 3.88 (SD=0.57) for peers; 3.82 (SD=0.57) for patients; 3.92 (SD=0.57) =0.59) for managers; 3.72 (SD=0.53) for hospital; 3.76 (SD=0.58) for physicians; and an overall EC of 3.82 (SD=0.51). In dichotomization, 83 (75.5%) nursing professionals perceived the EC as positive, and 27 (24.5%) as negative.

Note that among participants with burnout (n=11) and a perception of negative EC (n=27), n=5 of these participants were in both groups simultaneously. The associations between burnout dimensions and EC factors revealed a statistical association between burnout and EC, and between all its factors. The lowest EC mean values were found in the high DP and low PA groups, except for the EE factor, in which the high exhaustion group evaluated the EC more positively. The EC was classified as negative only in relation to burnout in the Physicians factor with a mean of 3.16 (IQR=0.67).

In the Qual survey, six interviews were conducted with the managers of covid-19 ICU at the UH; three at H1, one at H2 and two at H3. The information was organized from the textual analysis and priority categories were established based on the theoretical framework of burnout (EE; DP and PA) and EC (peers, physicians, managers, hospital, patients).

Next, in Chart 1, the joint-display^(16-17)^ presents the integrated findings of the study, highlighting the QUAN results of burnout and EC, as well as their associations with the reports extracted from the Qual analysis. Subsequently, for the inferences produced from each stage (QUAN+qual), meta-inferences are recommended. These results made it possible to expand knowledge about the association between burnout and EC, and its relationship with the organization of work in the covid-19 ICU in the perception of nursing professionals and managers of these units.

**Chart 1:** Joint-display of quantitative and qualitative inferences and meta-inferences of mixed methods about the relations of work organization, burnout and ethical climate in the covid-19 intensive care units of the university hospitals of the Brazilian Company of Hospital Services in Rio Grande do Sul during the covid-19 pandemic period, Santa Maria, Rio Grande do Sul, Brazil, 2022

1. MAIN TOPIC: Burnout and perception of the ethical climate in covid-19 ICU nursing professionals and the relationship with the organization of work from the perspective of managers of these units.			
**2. INTEGRATION: QUAN+qual**			
Burnout prevalence: high emotional exhaustion, n=33 (30%), high depersonalization, n=40 (36.4%), low professional achievement, n=27 (24.5%). Burnout: n=11(10%). Nurses: n= 5 (17,2 %) and Nursing technicians: n=6 (7,4%).			
Factor Concept (Olson, 1998)	EC Mean (SD)	Examples of participants’ speeches	Meta-inferences
Peers: It covers how supportive and transparent the relationships between colleagues are, reflecting on the quality of care.	3.88 (0.57)	1- The covid unit came, so we increased the number of employees to manage this new unit, many new employees started. (X2) 2- A nursing team very engaged in care, they didn’t go to the bathroom, didn’t have a snack, worked long shifts with no breaks, we left there exhausted, with emotional and physical fatigue, serving and managing the new routines; impotence generated great anguish... because the number of deaths was very high. (X3)	Convergences The Peers factor associated with the dimensions of burnout, especially in emotional exhaustion, reveals work relationships marked by differences in professional experience, a great number of new hires in the institutions and teamwork permeated by a difficult nursing care routine in a pandemic scenario with a high level of stress among people and exhaustion of professionals. The uncertainties caused by changes in the work organization process (new protocols) in covid-19 ICU influenced the emergence of professional exhaustion and the perception of the EC in this scenario.
Patients: Reflects how much information is shared between health professionals and patients	3.82 (0.57)	1- Arriving at the shift with a crowded unit and seeing there were few technicians, a colleague was crying. The atmosphere was always one of apprehension, patients walked in and left in black bags. We could only show a picture of the death, it wasn’t pleasant, but it was the alternative to show that their relative was in that bag. (X4)	The organization of work and the relationship with patients was marked by the instability surrounding covid-19, which caused tension, fear, stress and exhaustion (overload) of professionals.
Managers: They refer to the immediate leadership and general leadership of the organization, addressing aspects related to the respect and support from managers to nurses.	3.92 (0.59)	1- Some nursing technicians and nurses with morbidities; fear led to excessive care, causing conflicts in the team. (X1) 2- Although we had a very valid structure, there were things the unit needed, but nobody entered, it was shielded. (X5) 3- Working in an environment of death was the downside of covid-19. (X3) 4- The impotence was that of needing to do more, but not having the opportunity, those were endless weeks. (X6)	The Managers factor was related to the coordination and relationship with nursing professionals, revealing management in a scenario of uncertainties with new technical and administrative needs, lack of nursing workers given the sick notes or the greater demand for care, generating overload in the role of manager of the nursing team. The commitment of managers in this scenario was demonstrated with a positive EC mean, although with favorable elements to the occurrence of burnout.
Hospital: Presents the relationship between health professionals and hospital management, about the institution’s mission.	3.72 (0.53)	1- If the pandemic lasted longer, I don’t know if we would have financial, physical and mental conditions. (X4) 2- We needed someone from the care team to go to the meetings and inform about our covid-19 ICU needs. (X5)	The Hospital factor highlighted the need for a closer relationship between frontline professionals and institutional managers in order to assess and understand the work situation that constituted an environment of high emotional tension, mainly because of the work overload arising from the growing technical and material needs.
Physicians: Work climate between physicians and nurses. Shared relationship of knowledge and how ethical are the decisions made together.	3.76 (0.58)	1- Physicians without experience anchored in physicians with experience; sometimes there was mistrust, difficulties in making decisions, some cases of difficulty also in terms of professional respect between teams, causing stress; I guess because there were several teams working, so these conflicts always end up getting near me, I go there and talk to those responsible, together we improve work. (X1)	The Physicians factor showed elements of professional exhaustion in relation to trust and respect between physicians and nurses in decision-making, demonstrating the still distant relationship with physicians.

*EC - ethical climate; SD - standard deviation; n - number; qual - qualitative; QUAN - quantitative; ICU - intensive care units; X - participant; + - e.*

## DISCUSSION

The results made it possible to assess the relationship between burnout and EC, identifying lower EC means in the dimensions of high DP, low PA and presence of burnout, and a negative EC in the relation with burnout was evaluated only in the Physicians factor. In contrast, in the EE factor, the high exhaustion group evaluated the EC more positively, thus revealing a constructive behavior among nursing professionals, focused on coping with covid-19, even in the face of difficult work^(18-19)^.

The prevalence of burnout was 10%. The literature has studies of nursing professionals demonstrating an occurrence of burnout between 8% and 89%^(16,18)^. The subjects’ negative perception of the EC of 24.5% was evidenced. However, the overall average of the study for EC was 3.78, which is a positive evaluation. The findings of this study, both on burnout and EC, were equivalent to the results of other studies with nursing in other health care settings^(18-22)^.

Consequently, when connecting the findings with the testimonies of covid-19 ICU managers, regardless of adversities arising from coping with covid-19, health professionals and managers came together to build the best possible work environment with mutual help and team spirit. These are important aspects to minimize the deleterious effects of the pandemic on workers’ health^(22-23)^.

The values found in the dimensions of burnout and the perception of the EC corroborated by the testimonies of ICU managers show that the organization of work in the ICU during the pandemic influenced the work environment and workers’ health. Note that the understanding of the health and disease process has several aspects, such as the organization of work and healthcare resources, not being limited only to biological components for occupational illness^(23-24)^. Specifically in the context of covid-19, both nurses and managers, many also nurses in the covid-19 ICU, experienced several difficulties in the organization and dimensioning of teams, training and qualifications, resource management, and in the care for the mental health of professionals, who, in turn, faced an unknown agent, requiring the adoption of practices and performance of procedures that were unusual until then, and these constituted stress factors.

In this sense, covid-19 ICU nursing professionals from four hospitals in RS showed a prevalence of 54.9% of minor psychic disorders and 11.1% of burnout. These variables showed negative correlations with resilience at work, and evidenced that the impact of the pandemic interfered with the mental health of professionals. The authors also mention that this impact of the work context on professionals, when not managed, can result in greater professional exhaustion and both physical and psychological harm^(25)^. In the present study, considering the managers’ narratives, their confrontations and conflicts in the face of decision-making, compared to the results of burnout and EC in the perception of professionals, offered a greater understanding of the care and work process of nursing in the covid-19 ICU.

Finally, regarding the relationship between burnout and EC in the Physicians factor, evaluated by nursing professionals as negative, the eventual occurrence of conflicts between professionals in the medical and nursing areas emerges as a reflection of several factors involving the formation of the multidisciplinary health team and organizational structure issues. From this study, it became clear that nursing workers still consider their professional relationship with physicians as distant, even in this pandemic scenario, where medical decisions of patients could not be taken in isolation, as in many other places, given the peculiarities and variables involved in this context^(26-27)^.

It is also noteworthy that the covid-19 scenario presented variables with the power to influence this factor (physicians), such as: long work shifts, the high level of stress and workload, personal reasons, fear, insecurity and professional experience. Furthermore, health professionals (physicians, nurses and others) were surrounded by serious patients, suffering, despair, anguish, anxieties, nervousness, dilemmas and deaths, which were situations strongly intensified in the pandemic that could also interfere in the result^(26-27)^.

### Study limitations

The collection of the study occurred in a period of decrease in number of cases and reduction and closure of beds. These situations may have influenced the completion of the study instruments.

### Contributions to Nursing, Health or Public

The present study contributed to filling gaps in the literature on burnout and EC in nursing in the ICU within a scenario such as coping with covid-19.

## CONCLUSIONS

This study showed the presence of a 10% prevalence (n=11) of burnout and 24.5% (n=27) of negative EC among nursing professionals who worked in the covid-19 ICU of UH of the EBSERH in RS. Burnout was associated with EC, and both burnout and EC were related to tension, fear, stress and work overload that emerged from consequences of the organization and relationships of the nursing work of these professionals in the covid-19 ICU. Prophylactic actions such as continuing education in service may favor the minimization of harmful effects on the health of nursing workers, hence, they are recommended.

## Data Availability

https://doi.org/10.48331/scielodata.GCMDC2
